# Exercise counteracts fatty liver disease in rats fed on fructose-rich diet

**DOI:** 10.1186/1476-511X-9-116

**Published:** 2010-10-14

**Authors:** José D Botezelli, Rodrigo F Mora, Rodrigo A Dalia, Leandro P Moura, Lucieli T Cambri, Ana C Ghezzi, Fabrício A Voltarelli, Maria AR Mello

**Affiliations:** 1 São Paulo State University - UNESP, Department of Physical Education, Av: 24-A, 1515 Bela Vista. Zip code: 13506-900 Rio Claro, São Paulo, Brazil; 2 Federal University of Mato Grosso, Department of Physical Education, Fernando Corrêa da Costa Avenue, Boa Esperança. Zip code: 78060-900 Cuiabá, Mato Grosso, Brazil

## Abstract

**Background:**

This study aimed to analyze the effects of exercise at the aerobic/anaerobic transition on the markers of non-alcoholic fatty liver disease (NAFLD), insulin sensitivity and the blood chemistry of rats kept on a fructose-rich diet.

**Methods:**

We separated 48 Wistar rats into two groups according to diet: a control group (balanced diet AIN-93 G) and a fructose-rich diet group (60% fructose). The animals were tested for maximal lactate-steady state (MLSS) in order to identify the aerobic/anaerobic metabolic transition during swimming exercises at 28 and 90 days of age. One third of the animals of each group were submitted to swimming training at an intensity equivalent to the individual MLSS for 1 hours/day, 5 days/week from 28 to 120 days (early protocol). Another third were submitted to the training from 90 to 120 days (late protocol), and the others remained sedentary. The main assays performed included an insulin tolerance test (ITT) and tests of serum alanine aminotransferase [ALT] and aspartate aminotransferase [AST] activities, serum triglyceride concentrations [TG] and liver total lipid concentrations.

**Results:**

The fructose-fed rats showed decreased insulin sensitivity, and the late-exercise training protocol counteracted this alteration. There was no difference between the groups in levels of serum ALT, whereas AST and liver lipids increased in the fructose-fed sedentary group when compared with the other groups. Serum triglycerides concentrations were higher in the fructose-fed trained groups when compared with the corresponding control group.

**Conclusions:**

The late-training protocol was effective in restoring insulin sensitivity to acceptable standards. Considering the markers here evaluated, both training protocols were successful in preventing the emergence of non-alcoholic fatty liver status disease.

## Background

Metabolic syndrome, also known as syndrome X or insulin resistance syndrome, encompasses a spectrum of disorders, one of the most important of which is impaired glucose tolerance. These disorders include insulin resistance (with or without type 2 diabetes mellitus), hypertension, obesity, dyslipidemia and endothelial dysfunction [1]. The World Health Organization offers a definition of metabolic syndrome that includes any individual who shows diabetes or insulin resistance and two of the following symptoms: glucose intolerance, high waist/hip ratio, high serum triglycerides concentrations, low serum HDL cholesterol concentrations, high blood pressure or high urinary albumin excretion [2]. According to data from the 2000 census, about 47 million people living in the United States have metabolic syndrome [3]. It is estimated that the prevalence of metabolic syndrome in the United States is 24% in the adult population and between 50-60% in the population over 60 years of age [4].

The signs of metabolic syndrome have been induced in rats by prolonged feeding on a diet containing high amounts of fructose [5]. Rats fed this type of diet have been used as an experimental model of human metabolic syndrome [6,7], although they have not always expressed all signs of the syndrome, as shown by previous studies [8,9]. Non-alcoholic fatty liver disease (NAFLD) is emerging as an acknowledged component of metabolic syndrome. Markers of this condition, such as elevations in serum concentrations of aspartate aminotransferase transaminase (AST), alanine aminotransferase (ALT) and alkaline phosphatase (ALP), may be considered reliable predictors of the development of the syndrome [10].

Physical activity has been considered of great importance in the treatment of metabolic syndrome [11]. The intervention of exercise clearly improves glucose tolerance and reduces insulin resistance. However, exercise training requires procedures for controlling training intensity and volume. For this purpose, several protocols have been developed in the last decades. Many of these evaluation protocols use blood lactate concentrations due to their reliability in the measurement of the aerobic/anaerobic metabolic transition (the anaerobic threshold) during exercise and to their excellent metabolic response to physical training, a response that allows both the characterization of effort and the monitoring of training efficiency.

The anaerobic threshold (AT) is defined as the workload at which blood lactate begins to accumulate exponentially during exercise with progressive loads [12]. Theoretically, this threshold indicates the maximal lactate steady state [13]. The maximal lactate steady state (MLSS) is the highest blood concentration of lactate for which its output during exercise at a constant workload compensates for its input into circulation [14,15].

Studies on the metabolic effects of exercise training in animal models are sometimes criticized due to the lack of information about the intensity of effort performed by the animal during the exercise. Previously, our group developed experiment to determine the MLSS of rats during swimming [16]. This study enabled us to estimate the individual intensity of effort corresponding to the metabolic transition that occurred during the rats' swimming exercise.

Moreover, some studies verified the effect of physical activity in rats at different ages. Early training can prevent the NAFLD appearance. This may occur due to both the raise in energetic expenditure and the impairment in body weight gain and lipid storage, as well anti-oxidant mechanism improvement [17,18]. Otherwise, exercise training during the adulthood is able in counteracting several deleterious effects caused by occidental diets without changes in body weight and fat-free body mass [18,19].

The present study aimed to examine the effects of swimming exercise performed at the MLSS intensity started early or late in life on body weight, blood chemistry, insulin sensitivity and non-alcoholic fatty liver disease markers in rats fed on a fructose-rich diet used as a model of metabolic syndrome.

## Methods

### Animals and their Treatment

Forty-eight weaned male Wistar rats (28 days old) were used in the present study. The animals were kept in collective cages (four animals per cage) at 25 ± 1°C in a light/dark cycle of 12/12 hours and with ad libitum access to food and water. The experiments were performed at the Laboratory of Nutrition, Metabolism and Exercise of the Department of Physical Education of UNESP-São Paulo State University, Rio Claro, Brazil. The animals' body weight was recorded once a week throughout the experiment, and the area under the curve of body weight was calculated using Microsoft Excel 2007^® ^[20]. All experiments with animals were reviewed and approved by the Ethics Committee of the Herminio Ometto Foundation (UNIARARAS), Case Number: 068/2008.

### Diet treatment

As a model of metabolic syndrome, we used fructose-fed rats [5]. Animals fed on a balanced diet (AIN-93) [21] were used as a control. The composition of the diets is described in Table 1.

**Table 1:** Isocaloric diet composition

Components (g/Kg)	**Balanced**^**1**^	Fructose-rich Diet (66%)
Casein	202	202
Cornstarch	397	-
Dextrinized Cornstarch	130.5	-
Sucrose	100	27.5
Fructose	-	600
L-Cystine	3	3
Soybean oil	70	70
Mineral Mix (AIN-93GMX)^1^	35	35
Vitamin Mix (AIN-93GVX)^1^	10	10
Fiber	50	50
Choline Bitartarate	2.5	2.5

1. According to Reeves [18].

### Design and experimental groups

One week after weaning (28 days old), the animals were divided into 6 groups:

• Control (C): fed a balanced diet (AIN-93 G) and kept sedentary (untrained) until the end of the experiment;

• Control early trained (CET): fed a balanced diet and submitted to swimming training at the intensity equivalent to the MLSS from 28 days old until the end of the experiment;

• Control late trained (CLT): fed a balanced diet and submitted to swimming training at the intensity equivalent to the MLSS from 90 days old until the end of the experiment;

• Fructose (F): fed a fructose-rich diet and kept sedentary (untrained) until the end of the experiment;

• Fructose early trained (FET): fed a fructose-rich diet and submitted to swimming training at the intensity equivalent to MLSS from 28 days old until the end of the experiment;

• Fructose late trained (FLT): fed a fructose-rich diet and submitted to swimming training at the intensity equivalent to the MLSS from 90 days old until the end of the experiment.

### Adaptation to the water

Sedentary and both trained groups were first allowed to adapt to the water tank. The adaptation was performed for fifteen uninterrupted days in the same tank in which the exercise training was performed, with water temperature maintained at 31 ± 1°C [16]. The purpose of the adaptation was to reduce the stress of the animals without promoting the physiological changes that might arise from the physical training. The rats were initially placed in shallow water without workload for fifteen minutes during three consecutive days. The water level, the water exposure time and the overload sustained by the animals were subsequently increased. On the fourth day, the rats swam in deep water for two minutes, and swam for an additional two minutes each day until the tenth day of adaptation. On the eleventh day, the animals were submitted to swimming exercise for five minutes carrying a workload of 3% in relation to their body weight, with increases of five minutes every day. On the fifteenth day, the adaptation was concluded.

### Aerobic/anaerobic metabolic transition and evaluation of aerobic conditioning

Identification of the aerobic/anaerobic metabolic transition during swimming was performed using the MLSS protocol. In short, the animals were submitted to four swimming tests that supported constant and increasingly growing workloads relative to body weight. These tests were given at intervals of forty eight hours until the stabilization of blood lactate concentrations during exercise was no longer possible. Each test consisted of thirty minutes of continuous swimming supporting the workload, with blood collection by a small cut at the tip of the tail every five minutes to determine the concentrations of lactate. The blood lactate concentrations were then determined by a spectrophotometer [22]. The criterion used for stabilization was a difference less than or equal to 1.0 mM of blood lactate between the 10^th ^and 25^th ^minutes of exercise [16].

### Physical Training

The trained animals were submitted to swimming exercise in collective tanks containing water at 31 ± 1°C, one hour per day, five days per week, supporting a lead weight tied to the chest that created a workload equivalent to the individual aerobic/anaerobic metabolic transition identified by the MLSS test. At 28 days of age, the animals of CET and FET groups were evaluated on the MLSS to determine the exercise workloads. At 90 days of age, the animals of CET and FET groups were re-evaluated on the MLSS test in order to adjust the workloads. At this same point, the animals of CLT and FLT were subjected to the same test to identify the workloads used on training.

### Insulin Sensitivity

Insulin sensitivity was evaluated by the insulin tolerance test. The test consisted of a bolus injection of insulin (300 mU/kg body weight) followed by blood sample collections (for the measurement of glucose concentrations) from a cut at the tip of the tail before and 30, 60 and 120 minutes after the insulin injection. The serum glucose disappearance rate (Kitt) was calculated using the formula 0.693/*t*_1/2 _where *t*_1/2 _is the half-life of the process. The serum glucose half-life was calculated from the slope of a least-square analysis of serum glucose concentrations from 0 to 60 minutes after the subcutaneous injection of insulin, during this time, the glucose reduces linearly [20].

### Non-alcoholic fatty liver markers

As markers of non-alcoholic fatty liver disease, the serum activity of the transaminases aspartate aminotransferase (AST) and alanine aminotransferase (ALT), as well as and the total liver lipid concentration, were evaluated at the end of the experiment (120 days of age).

### Blood and tissue sample collection

Forty eight hours after the last *in vivo *evaluation, the animals were killed by decapitation. Blood samples were collected for serum separation, for the determination of the activity of hepatic transaminases AST and ALT, glucose, triglyceride and total-cholesterol concentrations by spectrophotometry using commercial kits (Laborlab^®^) [23]. The liver was also excised for the determination of the total lipid concentration [23].

### Statistics

The Shapiro-Wilk W test was used to verify the normality of the samples. The results were then subjected to a two-way analysis of variance (ANOVA). When necessary, the Bonferroni post hoc test was used. In all cases, the level of significance was set at 5%. The software used for the analysis was Statistica 7.0.

## Results

**Figure 1:**
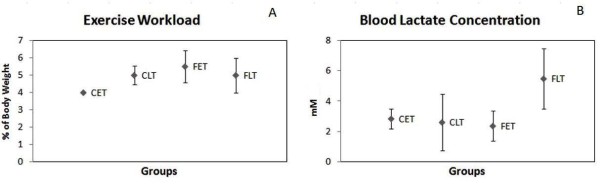
**Blood lactate concentration and workload values at the maximal lactate steady state of the animals during swimming exercise**. Results are expressed as the Mean ± SD. n = 8; C = Control Sedentary, CET = Control Early Trained, CLT = Control Late Trained, FET = Fructose Early Trained and FLT = Fructose Late Trained. Different letters indicate different values among groups p ≤ 0.05)

Figure 1 shows the stabilization values of blood lactate during the MLSS test and the workload equivalent to the MLSS during swimming exercise performed by the animals.

**Figure 2:**
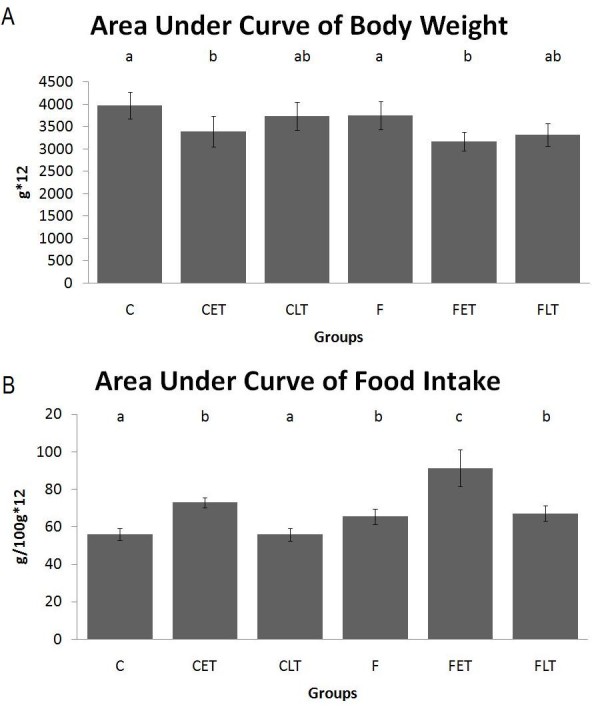
**(A) Area under body weight curve and (B) area under food intake curve of the animals during 12 weeks calculated by the trapezoidal method**. Results are expressed as the mean and standard deviation of eight animals per group. Results are expressed as the Mean ± SD. n = 8; C = Control Sedentary, CET = Control Early Trained, CLT = Control Late Trained, F = Fructose Sedentary, FET = Fructose Early Trained and FLT = Fructose Late Trained. Different letters indicate different values among groups (p ≤ 0.05).

Figure 2 shows the body weight evolution, the area under the curve of body weight evolution, food intake and the area under the curve of food intake of the animals during the 12 weeks of experiment. The early-trained groups (CET and FET) had a lower area under the curve of body weight when compared to the sedentary controls (C and F). The F group engaged in more food consumption in comparison with the C group. The early-trained animals (CET and FET) exhibited increased food intake compared with the other groups.

**Figure 3:**
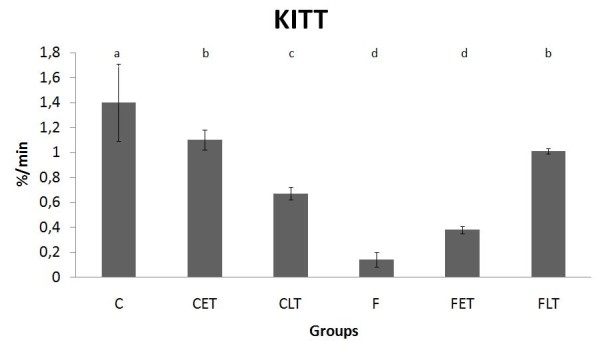
**Serum glucose disappearance rate (Kitt) during the insulin tolerance tests (ITT) at the end of the experiment**. Results are expressed as the Mean ± SD. n = 8; C = Control Sedentary, CET = Control Early Trained, CLT = Control Late Trained, F = Fructose Sedentary, FET = Fructose Early Trained and FLT = Fructose Late Trained. Different letters indicate different values among groups (p ≤ 0.05).

Figure 3 shows the serum glucose concentrations and the Kitt values during the insulin tolerance test. The sedentary fructose group (F) showed a lower Kitt value when compared with the sedentary control (C), indicating a lower insulin sensitivity. Physical training decreased the Kitt values in the control trained groups (CET and CLT), but the training increased Kitt values in the fructose-fed animals (FLT and FET).

The blood biochemistry variables are presented in Table 2. The fructose-trained groups (FLT and FET) had higher serum triglycerides values when compared with the corresponding controls (CET and CLT).

**Table 2:** Serum Glucose, Cholesterol and Triglycerides, at the end of the experiment

Groups/Parameters	C	CET	CLT	F	FET	FLT
**Glucose (mg/dl)**	112.9 ± 27.5	100.6 ± 36.5	96.3 ± 13.8	107.0 ± 24.6	88.1 ± 18	101.3 ± 16.6
**Total Cholesterol (mg/dl)**	90.4 ± 17.3	83.7 ± 13.2	92.7 ± 19.1	99.3 ± 24.3	83.7 ± 10.9	91.4 ± 16.5
**Triglycerides (mg/dl)**	150.4 ± 35.1^abc^	179.6 ± 32.6^a^	145.9 ± 40.3^ab^	173.0 ± 32.9^ab^	213.2 ± 40.2^b^	192.1 ± 36.3^c^

Results are expressed as the mean ± SD. n = 8; C = Control, CET = Control Early Trained, CLT = Control Late Trained, F = Fructose, FET = Fructose Early Trained, FLT = Fructose Late Trained. Different letters indicate different values among groups (p ≤ 0.05).

**Figure 4:**
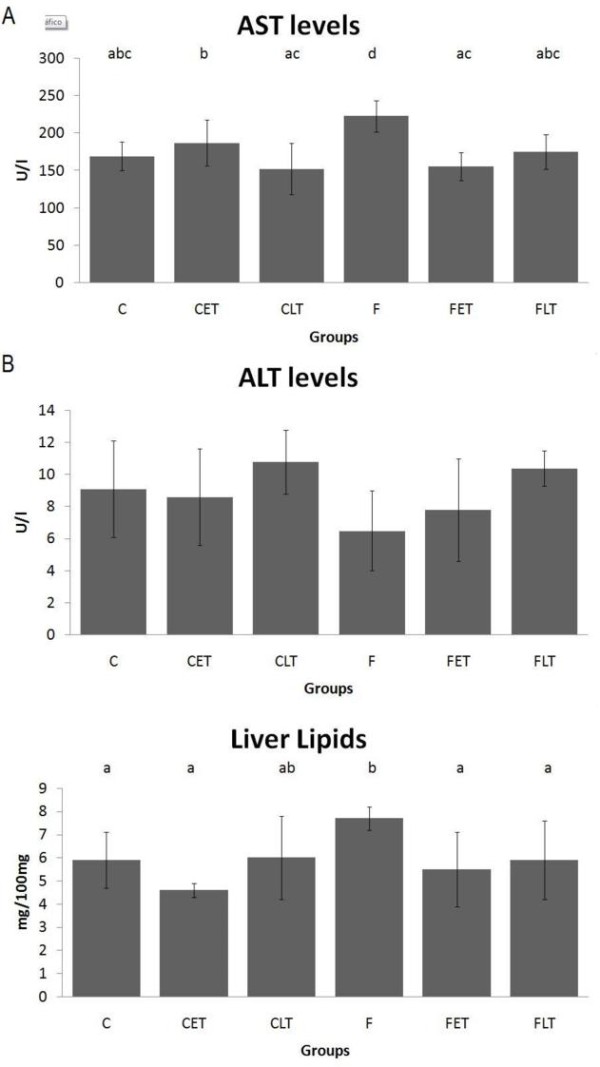
**(A) Serum Aspartate Aminotransferase [AST], (B) Serum Alanine Aminotransferase [ALT] and (C) liver lipids levels at the end of experiment**. Results are expressed as the Mean ± SD. n = 8; C = Control Sedentary, CET = Control Early Trained, CLT = Control Late Trained, F = Fructose Sedentary, FET = Fructose Early Trained and FLT = Fructose Late Trained. Different letters indicate different values among groups (p ≤ 0.05).

The analysis of serum transaminases and liver lipids is described in Figure 4. The animals of the CLT group showed lower AST (Figure 4A) concentrations when compared with the C group. The animals of the FLT and FET groups had lower AST (Figure 4A) concentrations when compared to the F group. No difference in the ALT (Figure 4B) values was observed among the groups. In addition, the F group showed high concentrations of liver total lipids (Figure 4C) when compared with the C, FLT and FET groups.

## Discussion

The increase in both fructose consumption and calorie intake, as well as the decrease in physical activity, have been identified as the main factors contributing to the growing numbers of obese and overweight individuals in many countries around the world [24,25] and experimental models have been widely used to analyze the metabolic effects of fructose-rich diets and exercise [26].

In this study, the body weights of animals fed a fructose-rich diet (F) were similar to that of those in the control group (C). The effects of fructose consumption on body weight seem to be dependent on rodent strain and on the administration period. Our results did not corroborate with other studies that used Sprague-Dawley rats and fructose-rich diets to induce obesity and overweight in rats [26,5]. The early-trained animals (CET and FET) exhibited a lower area under the curve of body weight when compared with their respective controls (C and F). The late-trained group showed no reduction in the area under the curve of body weight. On the other hand, the animals fed a fructose-rich diet showed an increase in food intake during the experiment compared with the respective control groups. Fructose does not stimulate insulin secretion by the pancreas and consequently does not stimulate leptin secretion by the adipose tissue. Leptin is a powerful satiety agent; animals fed a fructose-rich diet consumed larger amounts of food compared with animals fed a balanced diet [27]. On the other hand, the early-trained animals showed high food intake compared with the other groups (C, CLT, F and FLT), but had the lower body weight compared with the sedentary counterpart. Exercise alters the basal metabolism; consequently, the animals need more calories daily. Gaesser & Brooks [28] showed that physical exercise is responsible for 20% of the energy expenditure of sedentary individuals and can reach 40% in trained individuals. Furthermore, skeletal muscles can increase their energy expenditure by up to 100 times during exercise [28]. Moreover, the animals fed on fructose-rich diet presented higher food consumption if compared to control-diet. This result corroborate with previous studies [29,30] which used fructose to induce obesity in rats. Fructose consumption is not able to induce insulin secretion; consequently the leptin activation by insulin mechanism is not triggered. With lower levels of leptin, the central hungry control trends to down regulate the magnitude of adipocyte energy stores in adipose tissue, triggering the hungry mechanism [31].

The two exercise protocols here examined promoted different responses in body weight and food consumption. The early-training reduced body weight and increased food intake; the late protocol did not show these effects. In another studies, animals which performed early training protocol, had both physiological and morphological alterations, which lead to changes in fat deposition, fat distribution and lipid content in different tissues [17-19]. On the other hand, adult animals showed a consolidated body structure, and the alterations were found only at cell level and lipid content [18,19].

In addition to excess body weight, another important marker of metabolic syndrome is insulin resistance. The peripheral sensitivity to insulin was examined in this study through the serum glucose disappearance rate (Kitt) after exogenous insulin administration. As expected, the group fed a fructose-rich diet showed a reduction in insulin sensitivity. This finding agrees with several studies that show a sharp decrease in the sensitivity to insulin both in humans and in animals fed fructose-rich diets [32,33]. Bezerra et al. [9] demonstrated that two weeks of a high-fructose diet (66% of calories from fructose) did not alter the levels of IRS-1 and IRS-2 mRNA and, consequently, the number of IRS-1 and IRS-2 insulin receptors of the rats.

However, the mechanism of phosphorylation of these receptors showed a reduction of 72% in the hepatocytes and muscle fibers of the animals. Furthermore, other studies [34,35] reported that an increased level of triglycerides in liver or serum increases mitochondrial β-oxidation and stimulates a state of tissue inflammation. This, in turn, interferes with insulin sensitivity by increasing the concentrations of TNF-α and decreasing the concentrations of adiponectin cytokines.

Several studies have shown a direct relationship between levels of physical activity and insulin sensitivity [36,37]. Lower-circulating insulin concentrations and higher insulin sensitivity were exhibited in athletes when compared with sedentary individuals [38,39].

A single exercise session increases the insulin-mediated glucose utilization in normal or insulin-resistant subjects with a family history of type 2 diabetes, in obese individuals with insulin resistance and in patients with type 2 diabetes [40]. Physical training improves insulin sensitivity in healthy subjects, in obese non-diabetics and in diabetic patients (type 1 and 2) [40,41]. The CLT and CET animals showed a reduction in the Kitt values when compared with the C animals, whereas the animals of the sedentary fructose group (F) showed extremely low Kitt values; exercise-training (FET and FLT) counteracted this alteration. These late data are consistent with the literature, which indicates that physical exercise is a powerful weapon in combating insulin resistance [42,43]. According to these findings, both late- and early-exercise protocols had beneficial effects on insulin sensitivity measured by Kitt in fructose-fed rats.

Serum glucose concentrations were similar for the six groups at the end of the experiment. Glucose can be used for glycogen, lactate or even lipid synthesis. Fructose, on the other hand, does not have these properties. Fructose metabolism occurs in the liver, which can produce energy in the oxidative chain or provide carbon skeletons for lipid synthesis that spread to the blood stream. This characteristic makes fructose a highly lipogenic nutrient because it can only be stored as triglycerides [44]. Triglycerides generated by fructose play a key role in insulin resistance and, subsequently, in the increase of the concentration of circulating glucose. In the present study, however, no change in blood glucose was observed after fructose administration, probably due to the short period of the diet's administration. This finding corroborates previous studies by Fields et al. [45] and Catena et al. [46] in which no alteration in the blood glucose of fructose-rich rats was observed at the end of the experiments.

The maintenance of high-circulating triglycerides concentration causes an imbalance in lipid and carbohydrate oxidation [47]. This phenomenon can increase plasma glucose and, consequently, increase insulin production and secretion by the pancreas. Hepatic insulin resistance is caused by alterations in substrate 1 (IRS-1) and substrate 2 (IRS-2) of the insulin receptor. Protein N-terminal kinase (JNK) phosphorylates the IRS on serine, inhibiting its tyrosine phosphorylation, and thereby interrupting the glucose uptake process. These effects are at least partially the consequence of protein kinase C (PKC) and protein N-terminal kinase (JNK-1) activation, which are closely related to hepatic insulin resistance [35,48].

Therefore, changes in circulating triglyceride concentrations are the first and perhaps the main mechanism of hepatic steatosis induction. Lipid infiltration causes adverse effects to the liver, and the maintenance of high-circulating triglyceride levels can generate mild insulin resistance that may later develop into type 2 diabetes, dyslipidemia, atherosclerosis or central obesity, among other diseases [44].

Fructose absorption occurs rapidly in the small intestine by mechanisms that are still unclear. Unlike glucose, when it reaches the bloodstream it does not stimulate insulin secretion by pancreas beta cells. Still in circulation, it reaches the liver through the portal vein and is absorbed by the hepatocyte via the glucose transporters-5 (GLUT5) in an insulin-independent mechanism. An increased fructose concentration in the hepatocyte is followed by the use of fructose as an energy source or as the supply of carbon skeletons for lipogenesis. Unlike glucose, fructose cannot be stored as glycogen, a process that would provide carbon skeletons for lipid production through *de novo *lipogenesis. The key step in this process is in the conversion of glucose-6phosphate to fructose1-6phosphate. Although glucose conversion is modulated by phosphofructokinase (high levels of citrate can inhibits the phosphofructokinase action), fructose can continuously enter the glycolytic pathway with no destination. Because it cannot be converted into glycogen (an irreversible reaction), the fructose is converted into triglycerides, which are released into the blood stream, stored in the liver or in peripheral tissues or even connected to apolipoprotein B (apoB), thereby producing VLDL [47,48].

Serum triglycerides appeared significantly elevated in the FET group when compared with the CET group and in the FLT group when compared with the CLT group. During exercise at the MLSS intensity, the muscle metabolizes carbohydrates and lipids preferentially. The adaptation to exercise may have improved lipid oxidation and the availability of circulating lipids for energy production. In this case, instead of being stored in the adipose tissue and liver, these lipids were recruited for energy production. This finding can explain how the exercise attenuates fructose-induced insulin resistance, fat accumulation and liver damage.

Non-alcoholic fatty liver disease was diagnosed in this study through serum AST concentrations and the liver total lipid concentrations, which were higher in group F compared with groups C, FLT and FET. The studies of Lange [49] and Feldstein et al. [50] suggest that chronic exercise is an important tool in the prevention and treatment of hepatic steatosis, insulin resistance and circulating lipids concentrations regulation. The main effect of physical exercise on the hepatocyte is an increase in lipid oxidation, which reduces the levels of TG stored. Exercise also produces an increase in insulin sensitivity and in insulin-like growth factor (IGF-1), which are potent activators of liver regeneration and anabolism [9,49,50]. The increased serum triglycerides levels in the FLT and FET groups are in agreement with the above findings and can be explained by the adaptation of the animals to lipid metabolism, leading to a greater availability of this substrate in the bloodstream and, therefore, to a decreased liver infiltration.

## Conclusions

Physical exercise at the maximal lactate steady state was successful in reducing the body weight of early-trained animals. The late-training protocol was effective in restoring insulin sensitivity to an acceptable level in the animals. Finally, both training protocols were successful in preventing the onset of non-alcoholic fatty liver disease according to the markers we evaluated.

## List of Abbreviations

ALT: Alanine Aminotransferase; ApoB: Apolipoprotein B; AST: Aspartate Aminotransferase; AT: Anaerobic Threshold; IGF-1: Insulin-like Growth Factor 1; IRS-1: Insulin Receptor Substrate 1; IRS-2: Insulin Receptor Substrate 2; ITT: Insulin Tolerance Test; JNK: Protein N-Terminal Kinase; MLSS: Maximal Lactate Steady State; NAFLD: Non-alcoholic Fatty Liver Disease; PKC: Protein Kinase C; TG: Triglycerides; VLDL: Very Low Density Lipid.

## Competing interests

The authors declare that they have no competing interests.

## Authors' contributions

JDB was responsible for the experimental design, data collection, statistical analysis and preparation of the manuscript. RFM was responsible for the experimental design and for data collection. RAD, FAV and LTC were responsible for the data collection and the preparation of the manuscript. MARM was responsible for experimental design, coordination of research and preparation of the manuscript. All authors read and approved the final manuscript text.
